# Highly Accurate Prediction of Protein-Protein Interactions via Incorporating Evolutionary Information and Physicochemical Characteristics

**DOI:** 10.3390/ijms17091396

**Published:** 2016-08-25

**Authors:** Zheng-Wei Li, Zhu-Hong You, Xing Chen, Jie Gui, Ru Nie

**Affiliations:** 1 School of Computer Science and Technology, China University of Mining and Technology, Xuzhou 21116, China; zwli@cumt.edu.cn (Z.-W.L.); nr@cumt.edu.cn (R.N.); 2 Xinjiang Technical Institute of Physics and Chemistry, Chinese Academy of Science, Urumqi 830011, China; 3 School of Information and Electrical Engineering, China University of Mining and Technology, Xuzhou 21116, China; 4 Institute of Intelligent Machines, Chinese Academy of Sciences, Hefei 230031, China; guijie@ustc.edu

**Keywords:** evolutionary information, physicochemical characteristics, protein sequence, protein interactions, discriminative vector machine

## Abstract

Protein-protein interactions (PPIs) occur at almost all levels of cell functions and play crucial roles in various cellular processes. Thus, identification of PPIs is critical for deciphering the molecular mechanisms and further providing insight into biological processes. Although a variety of high-throughput experimental techniques have been developed to identify PPIs, existing PPI pairs by experimental approaches only cover a small fraction of the whole PPI networks, and further, those approaches hold inherent disadvantages, such as being time-consuming, expensive, and having high false positive rate. Therefore, it is urgent and imperative to develop automatic in silico approaches to predict PPIs efficiently and accurately. In this article, we propose a novel mixture of physicochemical and evolutionary-based feature extraction method for predicting PPIs using our newly developed discriminative vector machine (DVM) classifier. The improvements of the proposed method mainly consist in introducing an effective feature extraction method that can capture discriminative features from the evolutionary-based information and physicochemical characteristics, and then a powerful and robust DVM classifier is employed. To the best of our knowledge, it is the first time that DVM model is applied to the field of bioinformatics. When applying the proposed method to the *Yeast* and *Helicobacter pylori* (*H. pylori*) datasets, we obtain excellent prediction accuracies of 94.35% and 90.61%, respectively. The computational results indicate that our method is effective and robust for predicting PPIs, and can be taken as a useful supplementary tool to the traditional experimental methods for future proteomics research.

## 1. Introduction

Proteins are the building blocks of any living organism. Protein-protein interactions (PPIs) occur at almost all levels of cell functions in organisms [1]. Identification of PPIs is essential for deciphering molecular mechanisms and further providing great insight into various biological processes [2,3,4]. The analysis of disease-related PPIs can speed up new drug development and therapy breakthrough [5]. Recently, a variety of high-throughput experimental technologies, such as two-hybrid-based screens [6,7], protein chips [8] and spectrometric protein complex identification [9], have been proposed by investigators for the large-scale PPIs detection. However, these experimental techniques suffer from some inherent disadvantages such as significantly time-consuming, expensive, very low coverage and high false positive rate [10,11]. Therefore, it is highly desired to develop the efficient and accurate computational approaches to facilitate the prediction of novel PPIs [12].

In general, computational approaches for PPIs detection contain two critical steps: feature extraction and classification prediction [13,14]. Feature extraction is the foundation of the overall prediction process. If those extracted features are highly discriminative, they will facilitate the subsequent steps to significantly improve the success rate of PPIs prediction. In fact, numerous feature extraction approaches have been proposed to improve the performance of PPIs prediction. For example, Shen et al. developed a conjoint triad feature extraction method using only the information of protein sequence for predicting PPI and PPI networks [15]. Guo et al. adopted auto covariance of protein sequence to construct feature vector and obtained the promising prediction results [2]. Zhou et al. employed local descriptors to capture continuous and discontinuous binding patterns of protein sequences [16]. In addition, evolutionary-based features of protein sequences have also been widely used in PPIs prediction. Zahiri et al. extracted the evolutionary features based on position-specific scoring matrix (PSSM) of protein sequences [17]. Jia et al. incorporated seven physicochemical properties and wavelet transform to detect the interactions between proteins [4]. Although the aforementioned techniques have been demonstrated to be successful in PPIs analysis, they only utilized partial information of protein sequences (such as sequential information, or evolutionary-based information, or physicochemical characteristics). Considering the fusion of multi-class information may reveal some implicit correlations of protein sequences and are able to provide more discriminative information, we select four representative physicochemical characteristics integrated with evolutionary information based on PSSM of protein sequences to improve the prediction performance of PPIs.

Besides feature extraction, the following classification prediction is also critical. Many machine learning techniques have been employed for classification, such as support vector machine (SVM) [2,16,18], artificial neural network (ANN) [19,20], relevance vector machine (RVM) [21,22], collaborative filtering (CF) [23], weighted sparse representation [1,24] and ensemble classifier [4,25,26]. In this work, our newly developed discriminative vector machine (DVM) [27,28] classifier is used. To the best of our knowledge, it is the first time that the DVM model is applied to the field of bioinformatics. More specifically, we first use the position-specific scoring matrix (PSSM) to represent each protein sequence and calculate the corresponding PSSM probabilities. Second, each probabilistic residue product is calculated. Third, the autocorrelation coefficients are calculated and the final 160-dimensional vector for each protein sequence is constructed accordingly. Moreover, the proposed method is evaluated on the two different PPIs datasets: *Yeast* and *Helicobacter pylori* (*H. pylori*). The computational results show that our method yields good prediction accuracy. To further validate the performance of our method, it is compared with the state-of-the-art SVM classifier. Achieved results demonstrate that the proposed method is superior to SVM in prediction performance. Finally, comparisons between the proposed method and other previous works are implemented.

## 2. Results and Discussion

### 2.1. Performance of the Proposed Method on Yeast and Helicobacter pylori (H. pylori) Datasets

In this step, to minimize data dependence and avoid the over-fitting of the predicting model, fivefold cross-validation was adopted. As described in materials and methods section, the final *Yeast* dataset contains 11,188 protein pairs, half from the negative dataset and half from the positive dataset. Here four-fifths of the protein pairs (8950 protein pairs) respectively from the negative and positive dataset were randomly chosen to train the predicting model and the remaining one-fifths (2238 protein pairs) were employed for testing. To validate the robustness of the proposed approach, the random selection of training set and test set was repeated five times and five training sets and five test sets were obtained. Therefore, five predicting models on the *Yeast* dataset were generated accordingly. The processing method for the *H. pylori* dataset is the same as the one for the *Yeast* dataset. To facilitate the comparison between different experiments, the four physicochemical properties of protein sequence and parameters of the DVM predictor were set to the same for the *Yeast* and *H. pylori* datasets. The RBF function was chosen as the kernel function. The achieved results of the proposed method on the *Yeast* and *H. pylori* datasets are shown in Table 1 and Table 2.

When applying the proposed approach to the *Yeast* dataset, we got the prediction results of average accuracy (Acc), sensitivity (Sen), precision (Pre), and Matthews’s correlation coefficient (MCC) of 94.35%, 92.97%, 96.52%, and 89.07%, respectively. The corresponding standard deviations were 0.68%, 0.65%, 1.17%, and 1.56%. Similarly, the average values of accuracy, sensitivity, precision, and MCC on the *H. pylori* dataset reached 90.61%, 91.32%, 90.74%, and 82.79%. Their standard deviations were 1.55%, 1.48%, 1.81%, and 1.47%, respectively. The computational results indicate that the proposed method is successful in predicting PPIs.

From the results in Table 1 and Table 2, we can see that the DVM-based predicting model combining the four physicochemical properties with PSSM evolutionary information is accurate, effective and robust for the prediction of PPIs. The possible reasons of the excellent prediction performance lie in the highly discriminative hybrid features and the choice of the powerful DVM classifier. The proposed feature extraction method is novel and effective. As a representation of a protein sequence, PSSM not only retains the probability of any given amino acid at a particular position sequence but also holds sufficient prior evolutionary information. Apart from the use of PSSM, we also extracted four selected physicochemical attributes which also retain highly discriminatory information. By incorporating effective evolutionary-based information and physicochemical characteristics, the highly discriminatory features were formulated in the end.

### 2.2. Comparison with SVM-Based Method

To further evaluate the performance of the proposed method, we also constructed the state-of-the-art Support Vector Machine (SVM) classifier. Here, we used LIBSVM toolbox [29] as SVM classifier to carry out the prediction of PPIs. To be fair, the two predicting models adopted the same hybrid feature extracted from the *Yeast* dataset. A general grid search scheme was employed to optimize LIBSVM’s two parameters (regularization parameter C, kernel width parameter γ) and they (C, γ) were tuned to 0.7 and 0.3 respectively. Additionally, Gaussian function was chosen as the kernel function. For the DVM and SVM classifiers, all the input vectors were normalized in the range of [−1,1]. 

**Figure 1:**
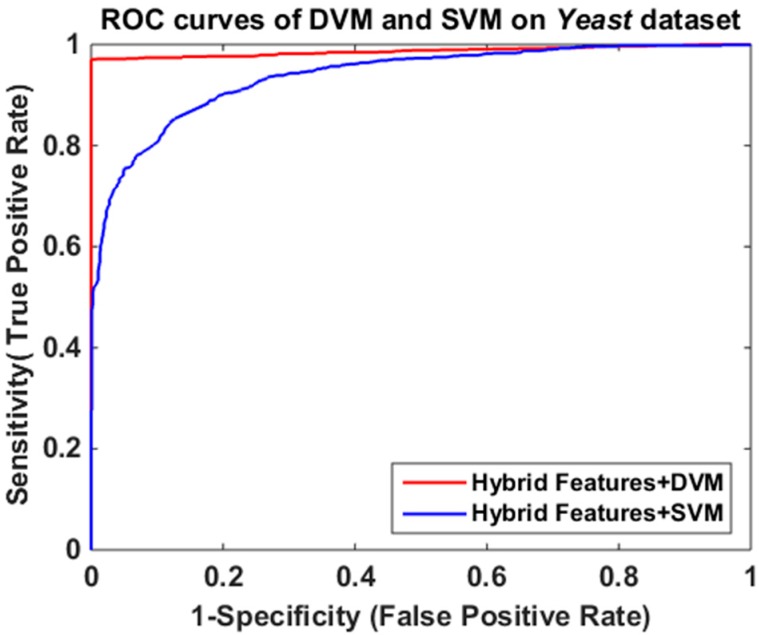
Comparison of receiver operating characteristic (ROC) curves between discriminative vector machine (DVM) and support vector machine (SVM) on *Yeast* dataset.

The final prediction results of the two methods are illustrated in Table 3 and the corresponding ROCs (receiver operating characteristic curve) are shown in Figure 1. From Table 3, the average prediction accuracy, sensitivity, precision and MCC of the SVM method attained 85.77%, 85.38%, 86.46%, and 75.65%, respectively. Meanwhile, the corresponding values based on DVM achieved 94.35%, 92.97%, 96.52%, and 89.07%, which indicate that our method is significantly better than SVM for predicting PPIs. Furthermore, as shown in Figure 1, the ROC of the DVM-based prediction model is superior to that of the SVM-based classifier. It obviously suggests that the proposed method is more effective and robust. There are two possible explanations to explain the results. (1) Based on k nearest neighbors (kNNs), the robust M-estimator and manifold regularization, DVM reduces the effect of outliers and overcomes the shortcoming of the kernel function being required to satisfy the condition of Mercer; (2) Although there are three parameters (*β*, *γ*, and *θ*) in DVM model, those parameters slightly affect the performance of DVM if they are adjusted in appropriate ranges. Therefore, the DVM-based model is more suitable for PPIs prediction than the SVM-based method.

### 2.3. Comparison with Other Methods

So far, numerous classification methods for predicting PPIs have been developed by investigators. To further validate the advantage of our approach, we compared the predictive performance of our method with other existing methods (as described in Table 4 and Table 5). The achieved results of fivefold cross-validation of different methods on the *Yeast* and *H. pylori* datasets are shown in Table 4 and Table 5. In Table 4, the prediction accuracy of other previous methods on the *Yeast* dataset varies from 75.08% to 93.92%, while the proposed method achieved higher value of 94.35%. Similarly, the sensitivity, precision and MCC of our method are also higher than those of other methods. Moreover, the corresponding standard deviations demonstrate the proposed method is stable and robust. Considering that ensemble classifier usually has better prediction effect than single classifier, although RF + PR-LPQ method has smaller standard deviations, our method is also considered as one of the most competitive computational methods for predicting PPIs. The similar results on the *H. pylori* dataset can also be found in Table 5. The highest prediction accuracy of six other methods is 89.47%, which is lower than the result (90.61%) of the proposed method. The same is true for precision, sensitivity and MCC. All prediction results in Table 4 and Table 5 indicate that the DVM classifier incorporating the evolutionary-based information and physicochemical characteristics can improve the prediction performance compared with the state-of-the-art methods. The high prediction performance of our method may contribute to the novel feature extraction method which extracts the highly discriminative information, and the use of DVM classifier which has been demonstrated to be robust and powerful [27].

## 3. Materials and Methods

### 3.1. Dataset

In this work, we evaluate the proposed method on the two high-confidence PPIs benchmarked datasets *Yeast* and *H. pylori* which are gathered from the publicly available Database of Interaction Proteins (DIP), version DIP_20070219 [34]. Those protein pairs in the datasets with less than 50 residues are excluded because they might be fragments. All protein pairs are aligned by using a multiple sequence alignment tool, cd-hit [35]. The protein pairs with too much sequence identity are generally considered to be homologous; so the pairs having ≥40% sequence identity are also removed. After above preprocessing, each dataset is divided into two subsets: negative dataset (non-interacting pairs) and positive dataset (interacting pairs). In the *Yeast* dataset, we select 5594 negative protein pairs as the negative dataset and 5594 positive protein pairs as the positive dataset. In the same way, 1458 negative protein pairs are selected to construct the negative dataset and 1458 positive protein pairs to form the positive dataset from *H. pylori* dataset. Therefore, the *Yeast* dataset consists of 11,188 protein pairs and *H. pylori* dataset includes 2916 protein pairs.

### 3.2. Feature Extraction

In this work, we aim to demonstrate that the perdition performance of PPIs can be improved by incorporating amino acids’ physicochemical properties and evolutional information. Although Taguchi and Gromiha held the viewpoint that physicochemical-based features do not carry important discriminative information [36], we believe that the combination of physicochemical properties with evolutionary information can provide highly discriminatory features for PPIs prediction. However, there are more than 544 physicochemical characteristics [37,38] . Fortunately, according to Gaurav Raicar et al., not all physicochemical properties play the same role for predicting PPIs [39]. Gaurav Raicar summarized the rank of physicochemical characteristics based on its frequency counts over all the datasets and a subset of them was identified. Here, through the extensive experiments, four physicochemical characteristics, including hydrophobicity (*H*), polarity (*P*), polarizability (*Z*), and van der Waals volume (*V*), are selected for the calculations. The numerical indices of the four physicochemical characteristics for the 20 amino acids are shown in Table 6.

Since the length of each protein sequence is different, the physicochemical characteristics and evolutionary-based information cannot merge directly. Based on pseudo amino acid composition (PseAAC) [40,41], we propose a novel feature extraction method which integrates position specific scoring matrix (PSSM) probabilities with the four physicochemical properties. PSSM is a representation of a protein sequence which defines the probability of any given amino acid occurring at a particular position in the sequence and carries the evolutionary information of protein sequence [39]. In this work, we adopt the position specific iterated BLAST (PSI-BLAST) tool to create PSSMs for all protein sequences of the *Yeast* and *H. pylori* datasets, via three iterations setting the E-value cutoff at 0.001 for the query protein sequence against multiple sequence alignment [10,42]. The PSSM P of a query protein sequence is a L × 20 matrix ($P=\left\{P_{i}^{j}\right\},$ i=1,2,…,L,j=1,2,…,20), where L is the length of the protein sequence and 20 denotes the 20 native amino acids. $P_{ij}$ is the score for the jth amino acid in the ith position of the given protein sequence [13]. The residue index $R_{m}$ for the mth physicochemical property is a column vector of 20 × 1 (as described in Table 6). Therefore, the probabilistic expression $F_{m}\left(m=1,2,\ldots ,4\right)$ of the residues about the mth physicochemical property can be defined as

(1) $$F_{m}=P\times R_{m}$$

where $F_{m}$ is a vector of size L × 1. It should be pointed out that the order of the amino acids in matrix *P* and vector $R_{m}$ must remain consistent. Then the hybrid features based on physicochemical characteristics and evolutionary information are calculated by using autocorrelation coefficients of the probabilistic expressions ($F_{m}$) of the protein sequence. The calculating formula is illustrated as

(2) $$V_{i}=\frac{1}{L-i}\overset{L-i}{\underset{j=1}{\sum }}\left(F_{m}^{j}-\mu \right)\left(F_{m}^{j+i}-\mu \right)$$

where $F_{m}^{j}$ is the jth probabilistic residue of $F_{m}$ on the mth physicochemical property in a protein sequence and *μ* is the average value of all $F_{m}^{j}\left(j=1,2,\ldots ,L\right)$. In this work, we use i = 1, 2, …, 40, thus producing 40 autocorrelation coefficients features to the mth physicochemical property. Therefore, each protein sequence is converted to a 4×40=160 dimensional feature vector.

### 3.3. Discriminative Vector Machine

Classification is a fundamental issue in pattern recognition field and there exist numerous classification algorithms for different recognition tasks. In this work, our newly developed discriminative vector machine (DVM) classifier is adopted in the classification. To the best of our knowledge, it is the first time that DVM model is applied to the field of Bioinformatics. DVM is a probably approximately correct (PAC) learning algorithm which can reduce the error caused by generalization and has strong robustness [27]. Given a test sample y, the first step of DVM is to find its k nearest neighbors (kNNs) to suppress the effect of outliers. The kNNs of y can be expressed by $X_{k}=\left[x_{1},x_{2},\ldots ,x_{k}\right]$, where $x_{i}$ is the ith nearest neighbor. For convenience, $X_{k}$ is also represented by $X_{k}=\left[x_{k,1},x_{k,2},\ldots ,x_{k,c}\right]$, where $x_{k,j}$ denotes the sample vector from the jth class. Then the objective of DVM is to solve the following minimization problem:

(3) $${}_{\;\;\beta_{k}}^{min}\sum_{i=1}^{d}\emptyset \left({\left(y-X_{k}\beta_{k}\right)}_{i}\right)+\delta ||\beta_{k}||+\gamma \overset{k}{\underset{p=1}{\sum }}\overset{k}{\underset{q=1}{\sum }}w_{pq}{\left(\beta_{k}^{p}-\beta_{k}^{q}\right)}^{2}$$

where ${\left(y-X_{k}\beta_{k}\right)}_{i}$ is the ith element of $y-X_{k}\beta_{k}$ and $\beta_{k}$ is denoted as $\left[\beta_{k}^{1},\beta_{k}^{2},\ldots ,\beta_{k}^{k}\right]$ or $\left[\beta_{k,1},\beta_{k,2},\ldots ,\beta_{k,c}\right]$, where $\beta_{k,i}$ is the coefficient from the ith class. ∅ is a robust M-estimator to improve the robustness of DVM. M-estimator is a generalized maximum likelihood operator proposed by Huber to estimate parameters under the cost function [43]. There are a variety of alternative robust estimators like Welsch M-estimator, MBA (Median Ball Algorithm) estimator and Cauchy M-estimator [44]. In this work, a robust Welsch M-estimator ($\emptyset \left(x\right)=\left(1/2\right)(1-exp\left(-x^{2}\right)$) is adopted to attenuate large error terms so that outliers would have a less impact on classification. $||\beta_{k}||$ is a norm of $\beta_{k}$ and the corresponding *l*2-norm is employed in our calculation. The last section of Equation (3) is the manifold regularization where $w_{pq}$ is the similarity between the pth and the qth nearest neighbor (NN) of y. In this work, $w_{pq}$ is defined as the cosine distance between the pth and the qth NN of y. Then the corresponding Laplacian matrix *L* can be depicted as

(4) L=D−W

where W is the similarity matrix whose element is $w_{pq}\left(p=1,2,\ldots ,k;q=1,2,\ldots ,k\right)$, D is a diagonal matrix whose ith element $d_{i}$ is the sum of $w_{iq}\left(q=1,2,\ldots ,k\right)$. According to Equation (4), the last section of Equation (3) can be denoted as $\gamma \beta_{k}^{T}L\beta_{k}$. Construct a diagonal matrix $P=\text{diag}\left(p_{i}\right)$ and its element $p_{i}\left(i=1,2,\ldots ,d\right)$ is:

(5) $$p_{i}=e^{-\frac{{\left({\left(y-X_{k}\beta_{k}\right)}_{i}\right)}^{2}}{\sigma^{2}}}$$

where σ is the kernel size which can be calculated in the following form:

(6) $$\sigma =\sqrt{(\theta \times {\left(y-X_{k}\beta_{k}\right)}^{T}\times \left(y-X_{k}\beta_{k}\right)/d}$$

where *θ* is a constant to suppress the effect of outliers. In this work, it is assigned to 1.0 as in the literature [45]. Based on the Equations (4)–(6), the minimization of Equation (3) can be converted to the following problem:

(7) $$\arg_{\;\;\;\beta_{k}}^{min}{\left(y-X_{k}\beta_{k}\right)}^{T}\;P\left(y-X_{k}\beta_{k}\right)+\delta ||\beta_{k}||{}_{2}^{2}+\gamma \beta_{k}^{T}L\beta_{k}$$

According to the theory of half-quadratic minimization, the global solution $\beta_{k}$ of Equation (7) can be solved by:

(8) $$\beta_{k}={\left(X_{k}^{T}PX_{k}+\delta I+\gamma L\right)}^{-1}X_{k}^{T}Py$$

After the related coefficients for each class are calculated, the test sample y can be identified as the ith  class if the residual $||y-X_{ki}\beta_{ki}||$ is the minimum distance.

(9) Ri=||y−Xkiβki||i   min,        i=1,2,…,c

As can be seen, DVM uses the robust M-estimator and manifold regularization to suppress the effect of outliers and improve its discriminatory ability; therefore, it has better robustness and higher generalization ability than kNNs. In this work, there are two classes in total to be identified: non-interacting protein pair (class 1) and interacting pair (class 2). If the residual $R_{1}$ is the minimum distance, the test sample y will be classified as non-interacting protein pair (class 1), or it will be identified as interacting protein pair (class 2). For three free parameters (δ, γ, θ) of the DVM model, it is time-consuming to directly search for their optimal values. It is gratifying that the DVM algorithm is so stable that all these parameters only affect the performance slightly if they are set in feasible ranges. Based on above knowledge and through grid search, the parameters δ and γ are set as 1 × 10^−3^ and 1 × 10^−4^ respectively. Just as described before, θ is a constant and is always set to 1 throughout the whole process. For large data set, the DVM classifier needs to spend relatively more time in finding the representative vector, so multi-dimensional indexing techniques can be adopted to speed up search process to a certain extent.

### 3.4. Procedure of the Proposed Method

**Figure 2:**
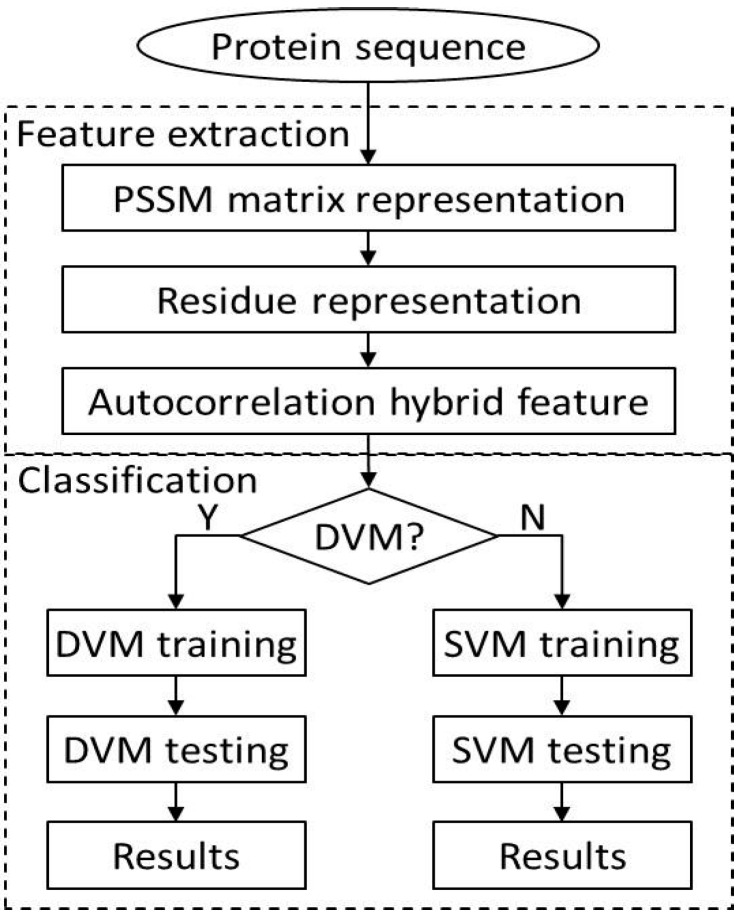
The flow chart of the proposed method.

In this study, the procedure of the proposed approach mainly consists of two steps: feature extraction and classification prediction. The feature extraction is also divided into three sub steps: (1) the PSI-BLAST tool is used to represent each protein sequence and the corresponding PSSMs are obtained; (2) Based on PSSM and physicochemical characteristics, each probabilistic residue $F_{m}$ is calculated; (3) Each autocorrelation correlation feature vector $V_{i}$ is established according to Equation (2). Similarly, classification prediction also includes two sub steps. (1) As described before, each dataset is divided into training set and test set. The training set is used to train the DVM model; (2) the trained DVM model is employed to predict the PPIs on the *Yeast* and *H. pylori* datasets and the performance of the algorithm is evaluated. Similarly, the SVM model is also constructed for predicting PPIs on the *Yeast* dataset. The flow chart of our proposed approach is illustrated as Figure 2.

### 3.5. Performance Evaluation

To evaluate the predictive performance of the proposed approach, four evaluation metrics, including the accuracy (*Acc*), sensitivity (*Sen*), precision (*Pre*), and Matthews’s correlation coefficient (*MCC*), were calculated. The concrete computational formulas can be formulated as follows:

(10) $$Acc=\frac{TP+TN}{TP+FP+TN+FN}$$

(11) $$Pre=\frac{TP}{TP+FP}$$

(12) $$Sen=\frac{TP}{TP+FN}$$

(13) $$MCC=\frac{\left(TP\times TN\right)-\left(FP\times FN\right)}{\sqrt{\left(TP+FN\right)\times \left(TN+FP\right)\times \left(TP+FP\right)\times \left(TN+FN\right)}}$$

where *FP*, *FN*, *TP* and *TN*, denote false positive, false negative, true positive and true negative, respectively. More specifically, *FP* is the number of non-interacting protein pairs that are falsely predicted to be interacting protein pairs, and *FN* denotes the number of interacting protein pairs that are falsely predicted to be non-interacting protein pairs. Similarly, *TP* represents the number of interacting protein pairs predicted correctly while *TN* stands for the number of non-interacting protein pairs predicted correctly. Furthermore, the Receiver Operating characteristic (ROC) curve is employed to evaluate the performance comparison between SVM and the proposed method.

## 4. Conclusions

In this work, we propose a novel computational method for predicting PPIs using the hybrid feature incorporating the evolutionary information and physicochemical characteristics of protein sequence. To minimize data dependence and avoid the over-fitting, five-fold cross-validation is adopted. When applied to the *Yeast* and *H. Pylori* datasets, the proposed method achieves good prediction accuracies of 94.35% and 90.61%, respectively. To further evaluate the performance of the proposed method, it is compared with SVM model and other previous works. The achieved results show that our proposed method is very competitive for predicting PPIs and can be taken as a useful supplementary tool to the traditional experimental methods for future proteomics research.

## Figures and Tables

**Table 1:** Fivefold cross validation results using the proposed method on *Yeast* dataset.

Testing Set	Acc (%)	Sen (%)	Pre (%)	MCC (%)
1	93.52	93.07	98.34	88.94
2	94.76	92.41	96.56	87.57
3	93.83	93.64	95.68	87.61
4	94.43	93.52	96.67	90.02
5	95.21	92.19	95.33	91.19
Average	94.35 ± 0.68	92.97 ± 0.65	96.52 ± 1.17	89.07 ± 1.56

Average accuracy (Acc), sensitivity (Sen), precision (Pre), and Matthews’s correlation coefficient (MCC).

**Table 2:** Fivefold cross validation results using the proposed method on *H. Pylori* dataset.

Testing Set	Acc (%)	Sen (%)	Pre (%)	MCC (%)
1	92.81	92.28	93.40	83.32
2	89.59	90.73	91.73	81.08
3	90.82	93.37	90.19	84.51
4	91.06	89.64	89.27	83.61
5	88.75	90.59	89.12	81.43
Average	90.61 ± 1.55	91.32 ± 1.48	90.74 ± 1.81	82.79 ± 1.47

Average accuracy (Acc), sensitivity (Sen), precision (Pre), and Matthews’s correlation coefficient (MCC).

**Table 3:** Fivefold cross validation results on *Yeast* dataset between our method and support vector machine (SVM).

Model	Testing Set	Acc (%)	Sen (%)	Pre (%)	MCC (%)
SVM	1	85.12	84.87	86.34	75.92
	2	86.16	83.91	85.36	74.99
	3	87.96	85.64	86.61	77.58
	4	85.42	85.80	88.67	75.02
	5	84.21	86.70	85.33	74.76
	Average	85.77 ± 1.41	85.38 ± 1.05	86.46 ± 1.36	75.65 ± 1.16
DVM	1	93.52	93.07	98.34	88.94
	2	94.76	92.41	96.56	87.57
	3	93.83	93.64	95.68	87.61
	4	94.43	93.52	96.67	90.02
	5	95.21	92.19	95.33	91.19
	Average	94.35 ± 0.68	92.97 ± 0.65	96.52 ± 1.17	89.07 ± 1.56

Average accuracy (Acc), sensitivity (Sen), precision (Pre), and MCC.

**Table 4:** Practical predicting results of different methods on the *Yeast* dataset.

Model	Testing Set	Acc (%)	Sen (%)	Pre (%)	MCC (%)
Guo [2]	ACC	89.33 ± 2.67	89.93 ± 3.68	88.87 ± 6.16	N/A
	AC	87.36 ± 1.38	87.30 ± 4.68	87.82 ± 4.33	N/A
Yang [30]	Cod1	75.08 ± 1.13	75.81 ± 1.20	74.75 ± 1.23	N/A
	Cod2	80.04 ± 1.06	76.77 ± 0.69	82.17 ± 1.35	N/A
	Cod3	80.41 ± 0.47	78.14 ± 0.90	81.66 ± 0.99	N/A
	Cod4	86.15 ± 1.17	81.03 ± 1.74	90.24 ± 1.34	N/A
You [25]	PCA-EELM	87.00 ± 0.29	86.15 ± 0.43	87.59 ± 0.32	77.36 ± 0.44
Wong [26]	RF + PR-LPQ	93.92 ± 0.36	91.10 ± 0.31	96.45 ± 0.45	88.56 ± 0.63
Proposed Method	DVM	94.35 ± 0.67	92.97 ± 0.51	96.52 ± 0.57	89.07 ± 1.30

N/A—Not applicable.

**Table 5:** Practical predicting results of different methods on the *H. Pylori* dataset.

Model	Acc (%)	Sen (%)	Pre (%)	MCC (%)
Nanni [31]	83.00	86.00	85.10	N/A
Nanni [32]	84.00	86.00	84.00	N/A
Nanni and Lumini [33]	86.60	86.70	85.00	N/A
You [25]	87.50	88.95	86.15	78.13
Martin [18]	83.40	79.90	85.70	N/A
Wong [26]	89.47	89.18	89.63	81.00
Proposed Method	90.61	91.32	90.74	82.79

N/A—Not applicable.

**Table 6:** Numerical indices of the four physicochemical characteristics for the 20 amino acids.

Amino Acid Name	Hydrophobicity	Polarity	Polarizability	van der Waals Volume
Alanine	0.61	8.1	0.046	1.00
Arginine	0.60	10.5	0.291	6.13
Asparagine	0.06	11.6	0.134	2.95
Aspartic Acid	0.46	13.0	0.105	2.78
Cysteine	1.07	5.5	0.128	2.43
Glutamine	0.0	10.5	0.180	3.95
Glutamic Acid	0.47	12.3	0.151	3.78
Glycine	0.07	9.0	0.000	0.00
Histidine	0.61	10.4	0.230	4.66
Isoleucine	2.22	5.2	0.186	4.00
Leucine	1.53	4.9	0.186	4.00
Lysine	1.15	11.3	0.219	4.77
Methionine	1.18	5.7	0.221	4.43
Phenylalanine	2.02	5.2	0.290	5.89
Proline	1.95	8.0	0.131	2.72
Serine	0.05	9.2	0.062	1.60
Threonine	0.05	8.6	0.108	2.60
Tryptophan	2.65	5.4	0.409	8.08
Tyrosine	1.88	6.2	0.298	6.47
Valine	1.32	5.9	0.140	3.00
